# When income buffers extreme weather: Impacts on women’s mental health in informal settlements

**DOI:** 10.1371/journal.pclm.0000720

**Published:** 2026-04-10

**Authors:** Samantha C. Winter, Laura Johnson, Ebuka Ukoh, Hailey Hansen, Haley Brown, Manuela S. Velasquez, Kianna Stamps, Lena Moraa Obara, Christine Musyimi, Malika Ali, Susan S. Witte

**Affiliations:** 1 School of Social Work, Columbia University, New York, New York, United States of America,; 2 School of Social Work, Temple University, Philadelphia, Pennsylvania, United States of America,; 3 Scripps Institution of Oceanography, University of California San Diego, La Jolla, California, United States of America,; 4 African Institute of Mental and Brain Health, Nairobi, Kenya

## Abstract

Climate change threatens mental health, especially for the more than one billion residents in informal settlements worldwide. Utilizing longitudinal data collected from women in households in two of Nairobi’s largest informal settlements, we examined the mental health impacts of extreme weather and moderating effects of income. Eighteen monthly surveys (September 2022–February 2024) captured experiences of heat, cold, drought, heavy rain, and flooding alongside symptoms of anxiety and depression. Heat, cold, and drought were associated with increased anxiety and depression while heavy rain reduced symptoms and flooding showed no significant association. Critically, income moderated the effects of heat and drought. We identified income thresholds at which heat and drought were no longer significantly associated with symptoms of depression and anxiety. While the income thresholds identified may not apply to all informal settlements, the same approach can help other communities develop locally-grounded guidelines for financial planning to reduce climate-related health risks and inequities. For example, women in households earning below KES 11,000 (~US$87) experienced significant mental health burdens during extreme heat while those with higher incomes did not. These findings suggest that financial vulnerability exacerbates climate-related mental health risks in these communities; however, finance-based interventions—such as forecast-based cash transfers, resilience grants, and community-centered employment programs—could buffer the psychological impacts of climate extremes while strengthening adaptation and reducing inequities.

## Introduction

Climate change is expected to have a profound impact on psychological well-being and mental health [1–5], especially for women [1,6] and those living in ecologically sensitive areas like floodplains, geographically hazardous zones, and wetlands [7]. Research documents numerous mental health outcomes associated with climate change and extreme weather, including increased despair, fear, helplessness, anxiety, depression, suicidal ideation, post-traumatic stress disorder (PTSD), decreased sense of self, identity loss, and grief [7]. Residents of climate vulnerable communities—those most susceptible to, or least able to cope with, the adverse effects of climate variability and extremes [8]—face particularly high risk. This is especially true in informal settlements, where residents are disproportionately exposed to extreme weather events (EWEs) like floods, landslides, heavy rains, droughts, sea-level-rise and heatwaves [6,9–15]. These climate-related threats intersect with structural inequalities—including political, social and economic marginalization, [15–19] stateled threats and evictions [16,18–20], and limited provision of necessary services [16,19,21,22]—to produce elevated rates of mental health challenges [23–30]. Gendered roles and systemic inequities further compound these risks. Women in these settlements experience greater vulnerability to extreme weather and have fewer resources, particularly economic, to cope or adapt [6,13,14,31].

Informal settlements house over a billion people, globally, including 51.3% of sub-Saharan Africa’s urban population [32]. They are characterized by non-durable housing, inadequate access to essential services, overcrowding, and insecure tenure [33]. These areas often lack clean water and sanitation and adequate waste management, and are located in high-risk zones such as flood plains or steep slopes with sparse vegetation, contributing to health challenges [6,9–12,16,34–37]. Importantly, residents also face poverty, lack of land tenure and legal recognition, and limited access to formal labor markets [10,15,38–40]. While socioeconomic resources, including adequate income, assets, and savings, can serve as critical buffers against negative impacts of climate-related extreme weather by enhancing coping capacity, expanding opportunities for recovery and adaptation, and strengthening overall resilience [10,38,41], many residents of informal settlements face chronic shortages of these resources [6,38,42–45].

The mental health burden is also especially high in informal settlements, with elevated rates of depression and anxiety, especially among women [24,46–49]. Research has attributed these mental health challenges to a combination of factors, including higher exposure to extreme weather; community and interpersonal violence; socioeconomic disadvantages such as poverty, reliance on informal work, and food insecurity; sub-standard living conditions; and lack of access to basic services like water and sanitation [18,24,31,46,47,49–52]. Household resources—including income, employment stability, and overall economic security—are prominent determinants of residents’ mental health [6,15,40,44,45,53,54]. However, these resources are highly vulnerable to climate change [3,10,55,56]. EWEs and disasters frequently disrupt income sources, reduce savings, and limit residents’ ability to work, particularly for those relying on informal work [13,15,40,42,44,57–59]. They can also increase household expenditures, debt, and responsibilities (e.g., home repairs, emergency supplies, healthcare, and dependent care) [14,15,31,42,44,54,57], further compounding mental health burdens.

Women in informal settlements are especially vulnerable to the mental health impacts of extreme weather [6,13,14,31,60]. As primary caregivers and informal workers, women in these communities depend heavily on access to food, water, and energy [6,14,40,60,61]–resources increasingly jeopardized by extreme weather. For example, women are often responsible for collecting water for cleaning and cooking [6,13,14,40,61]. Additionally, most women rely on informal labor, such as domestic work or small-scale vending, which often requires access to essential resources like water and food [15,18,40,62–64]. During EWEs such as droughts or extreme heat, access to water and food is variable, hindering women’s ability to complete these activities [14,56,65]. Scarcity during droughts or heatwaves disrupts caregiving and paid work, while price surges further increase stress [6,14,40]. Due to structural gender inequities, women in these settlements also typically earn less than men, hold fewer assets such as land, and are disproportionately employed in low-paid and insecure work [6,13,15,18,40,62].

Research suggests that socioeconomic resources can build climate resilience and buffer against poor mental health [10,38,41]. In fact, asset transfer programs and savings groups have been highlighted as promising strategies in informal settlements [10,45]. Presumably, adequate financial resources may also better position women to cope or adapt with climate-related extreme weather. However, little research has examined whether access to financial resources can buffer the mental health effects of climate-related extremes for women or how much is required.

To address this gap, we used longitudinal data from a probability sample of 800 women in Nairobi’s Kibera and Mathare settlements to examine (1) the impact of different types of extreme weather on women’s mental health and (2) whether and at what thresholds monthly income can moderate this relationship.

## Methods

### Study description

Data for this study were taken from a longitudinal investigation of the direct and indirect effects of extreme weather on mental health in a probability sample of women living in two large informal settlements—Mathare and Kibera—in Nairobi, Kenya. Monthly surveys were collected at the household level between September 01, 2022 and February 28, 2024. This study uses data from the full 18 waves of surveys.

### Study sites

Kibera and Mathare are two of the largest informal settlements in sub-Saharan Africa. The approximate population of Kibera and Mathare are likely around 450,000 [66–68] and 250,000, respectively [63,69]. These settlements are the two most densely-populated areas in Kenya. Kibera has a population density of 15,311 people per square km and Mathare has a population density of 68,940 people per square km [70]. Kibera and Mathare are characterized by high levels of poverty; overcrowding; inadequate access to basic services like water, sanitation, electricity, and solid waste management; and significant environmental challenges [71].

The settlements are situated in lowlands along Nairobi’s major rivers, exposing residents to annual flooding and mudslides during heavy rains [72,73]. Poor drainage systems, inadequate solid waste management, and poorly constructed housing exacerbate the impact of EWEs [74]. In addition, these settlements experience significantly higher temperatures than surrounding areas due to their densely built environment and lack of vegetation, leading to frequent heatwaves [75,76].

Over 90% of residents in these informal settlements do not own the land on which their homes are built, which limits their ability to invest in long-term, climate-resilient infrastructure [77]. Additionally, most residents in informal settlements in Nairobi rely on informal economies for income, such as selling fruits and vegetables or mobile phone airtime and basic household necessities or casual labor ‘gigs’ such as washing clothes and housework [39,63].

### Study sample

A probability sample of 800 women residing in Kibera (n = 400) and Mathare (n = 400) was obtained using a sampling approach that was previously implemented by the authors [78]. OpenStreetMap (.osm) was used to acquire settlement boundaries. In ArcGISPro version 3.0, polygon layers were added to satellite imagery to verify these boundaries. Once verified, a fishnet grid was superimposed on the settlement polygon layers. Each grid cell measured 9 square meters (3m × 3m)—the approximate size of a mud or tin house or a room in a high-rise. A random selection function was used to identify 400 grid cells for sampling in each informal settlement area. Subsequently, GPS coordinates for 50 random grid cells were loaded onto each of 16 community data collector’s (CDCs) tablets. CDCs used Google Earth and Google Maps on their tablets to navigate to the nearest household corresponding to each GPS coordinate.

Recruitment took place between September 1, 2022 and October 7, 2022. We approached 863 households, with 63 women not eligible or declining to participate (43 in Kibera and 20 in Mathare). Reasons for declining included anticipated relocation, lack of interest, and skepticism about research. The quasi-random “last birthday” technique [79] was used to identify one woman in households with more than one eligible woman. Eligibility criteria required participants to be at least 18 years of age, proficient in either English or Swahili, and residents of the informal settlement for at least 6 months (i.e., not visitors or temporary residents). Since Swahili is the lingua franca in informal settlements, limiting study participation to Swahili or English speakers did not affect resident participation.

### Ethical statement

This study involved human participants. It was approved by the Columbia University Institutional Review Board (IRB reference number: AAAU0353) and the Kenya Medical Research Institute (KEMRI reference number: KEMRI/RES/4476). In addition, national-level approval was obtained from the National Commission for Science, Technology and Innovation (NACOSTI reference number: NACOSTI/P/22/19316). The studies were conducted in accordance with local ethical and institutional requirements. All participants provided written informed consent prior to participation. Minors were not included in the study.

### Data collection

Household-level surveys were conducted monthly by trained CDCs who are also women residents of Kibera (8) and Mathare (8). CDCs underwent training for about 120 hours focused on principles of ethical research with human participants, quantitative data collection, study instruments, GIS data collection, surveying, informed consent, study protocols, and the World Health Organization’s (WHO) ethical and safety recommendations for research concerning violence against women and sensitive topics [80]. In adherence to these guidelines, researchers, local collaborators, and CDCs also established safety protocols to be enacted if a participant reported instances of violence and/or adverse mental health outcomes. All participants provided written consent following the informed consent process, during which CDCs provided study information and participants were given opportunities to ask questions about the study and their participation. The 60–90-minute survey was conducted in participants’ homes and administered using tablets.

### Measures

**Anxiety** was measured using the General Anxiety Disorder Score (GAD-7). The GAD-7 consists of seven items measuring the frequency of symptoms of anxiety in the past two weeks on a four-point Likert scale ranging from ‘0 = never” to ‘3 = nearly every day’. This measure has been widely used around the world [81]. Cronbach’s alpha is 0.88 in this sample. Standard thresholds for anxiety on the GAD-7 scale suggest that individuals with scores below five experience minimal anxiety. Scores between five and nine indicate mild anxiety, those between 10–14 indicate moderate anxiety, and those above 15 indicate moderately severe to severe anxiety [81].

**Depression** was measured using the Patient Health Questionnaire Score (PHQ-9). The PHQ-9 consists of nine items measuring the frequency of symptoms of depression in the past two weeks on a four-point Likert scale ranging from ‘0 = never” to ‘3 = nearly every day’. Cronbach’s alpha is 0.87 in this sample. Standard thresholds for depression on the PHQ-9 scale suggest that individuals with scores below five experience minimal depression. Scores between five and nine indicate mild depression, those between 10–14 indicate moderate depression, and those above 15 indicate moderately severe to severe depression [82].

#### Experiences of extreme weather.

Participants were asked whether they or members of their household had experienced any EWEs in the past month, defined as an event that was considered uncommon/more extreme for that place and time of year. If participants responded “yes,” they were then asked to indicate, from a checklist of possible EWEs, each type of event experienced—including extreme heat, cold, floods, heavy rainfall, drought, or other (specify)—along with the date(s) on which these events occurred. Participants could report additional events not included on the list.

We chose to focus on experiences of extreme weather rather than using meteorological data and pre-defined thresholds of extremes. This choice is based on findings from recent research that suggests meteorological data fail to capture the fine-scale variations in exposure experienced by residents of informal settlements [83]. One study carried out in Kibera and Mathare showed that residents experience extreme weather at thresholds much lower than those defined using meteorological data [78]. Self-reported weather experiences can offer a more direct insight into the lived exposure that impacts mental health [84]. Research examining the relationship between objective meteorological measures and subjective perceptions of weather phenomena has revealed that the correlations between established meteorological thresholds and individuals’ interpretations of “extreme weather” are often weak—suggesting that reliance solely on data from weather stations might lead to an oversight of socially and contextually significant experiences related to extreme weather [85].

#### Monthly income.

Participants in this study were asked to specify how much their household earned from each of the following sources: salary from employment, fees from consulting, payments from working gigs (e.g., washing clothes, cleaning, construction, sex work), profits from business/selling goods, other work, gifts from friends/family/other people, loans from friends/family/other people, material gifts or contributions (e.g., sugar, soap, flour, cooking oil), other money from friends/family/other people, money received from table banking/microfinance groups, loans from microfinance groups, material gifts or contributions from self-help groups, other money from self-help groups, money from harambees/contributions, or money from any other sources. Women were asked to specify amounts and the frequency of payment (e.g., daily, weekly, monthly, or lump sum/one-off). If women specified a daily rate or weekly rate, the amount was multiplied by 30.5 days or 4.5 weeks, respectively. Women were also allowed to select an option for “don’t know” if they were unsure of the values their household received in each category. Values of “don’t know” were treated as missing values and imputed at a later stage (see multiple imputation). All of the amounts were then added together to create a monthly total. After inspecting for outliers, we removed values that were greater than three times the standard deviation from the mean.

#### Survey wave.

Given the longitudinal nature of the data, a survey wave, ranging from ‘0 = baseline’ to ‘17 = follow-up 17’ was included.

#### Covariates and potential confounders.

Four time-invariant demographic variables including a three-category education variable (less than primary, completed primary, and completed secondary), a continuous age variable, a binary (yes/no) married variable, and a binary (Kibera/Mathare) settlement variable, were included as covariates. Two additional time-varying covariates were also included: a binary (yes/no) “partner worked in the past month” variable and a binary (yes/no) “participant worked or did something for money or other payments in the past month” variable.

A binary past-month intimate partner violence (IPV) variable and a food insecurity score were also included as important factors associated with mental health in informal settlements [24,86]. Past-month IPV was measured using the WHO-VAW CORR 5 [87], which includes 12 items capturing experiences of psychological, physical, and sexual violence. If a participant answered ‘yes’ to having experienced any type of IPV, she received a value of ‘1’ on the binary past-month IPV variable; if she answered ‘no’ to all 12 items, she received a ‘0’. Food insecurity was measured using the Household Food Insecurity Scale (HFIAS) [88]. The HFIAS consists of nine items assessing anxiety about food supply, insufficient food quality, and insufficient food intake over the past four weeks. Each item is first asked as an occurrence (yes/no), followed by a frequency-of-occurrence response (rarely, sometimes, or often). Responses are scored from 0 to 3, and summed to create a total food insecurity score ranging from 0 to 27, with higher scores indicating greater food insecurity.

### Analysis strategy

Analyses were guided by the following research questions: (RQ1) What are the associations between experiencing extreme weather and women’s anxiety and depression? (RQ2) In communities where finances are particularly tight, can higher monthly incomes moderate the effect of experiencing extreme weather on women’s anxiety and depression? (RQ3) If so, does the size of monthly income matter? All analyses were carried out in Stata 16.1.

#### Handling missingness.

Missingness across variables in this study was minimal (between 0.00–5.25%). We chose, however, to use multiple imputation to handle missingness because we had “don’t know” responses for the monthly income variables, which added additional missingness up to 7%. We used Multiple Imputation by Chained Equations (MICE) in Stata to address missing data. Fifteen imputed datasets (m = 15) were generated. The imputation model included all variables used in the primary analysis. The following fully observed variables were used as predictors: participant ID, survey wave, settlement, and birthyear. Appropriate models were specified based on variable type (e.g., linear regression for continuous variables, predictive mean matching for income variables, logistic regression for binary variables, ordinal regression for ordinal variables). Subsequent analyses were performed using mi estimate. Parameter estimates and standard errors were combined using Rubin’s rules.

We ran descriptive statistics on the multiply imputed data for all time-varying and time-invariant variables used in the models, with estimates pooled across the 15 imputations. To answer RQ1, we used mixed-effects models to account for the nested structure of the data. Participant ID was modeled as a level-2 grouping variable, allowing for random intercepts to account for individual differences. We also specified random slopes for survey wave, permitting each participant to have their own trajectory across time. We ran ten separate models for each type of extreme weather (extreme heat, cold, rain, flood, and drought) and each outcome (anxiety and depression). Fixed effects included experiences of extreme weather, monthly income, covariates, and potential confounders. To address RQ2, we added a moderating effect for experiences of extreme weather by monthly income to the models we used to address RQ1. Again, we ran 10 separate models for each type of extreme weather and each outcome (anxiety and depression). Finally, to answer RQ3, we used Stata’s margins commands to examine the marginal effect (dydx) of extreme weather on anxiety and depression at various levels of monthly income (KES 0–50,000 at increments of KES 1,000) for each type of extreme weather with a significant moderating effect. This allowed us to identify income thresholds at which the effect of extreme weather on anxiety and depression was statistically significant. Predicted values for anxiety and depression across income levels were also calculated for those who did and did not experience extreme weather and plotted to aid in the interpretation of the interaction.

To check normality assumptions of the mixed-effects models, standardized residuals were calculated and plotted on Q-Q plots. Mixed-effects models are robust to moderate departures from normality [89,90]; however, visual inspection of residuals indicate approximate normality with minor deviations in the tails.

## Results

### Participant characteristics

Table 1 presents pooled medians, IQRs, and proportions for time-invariant covariates. The median age of participants was 34 years (IQR = 27–42). Approximately one-quarter of the 800 participants had less than a primary education; just under 48% had completed primary, but not secondary education; and over 27% had completed secondary education. Slightly more than half (52%) of participants had partners who worked, and nearly 79% of the participants worked. Both partner and participant work were measured in the past 30 days; so, these percentages vary across time.

**Fig 1.**
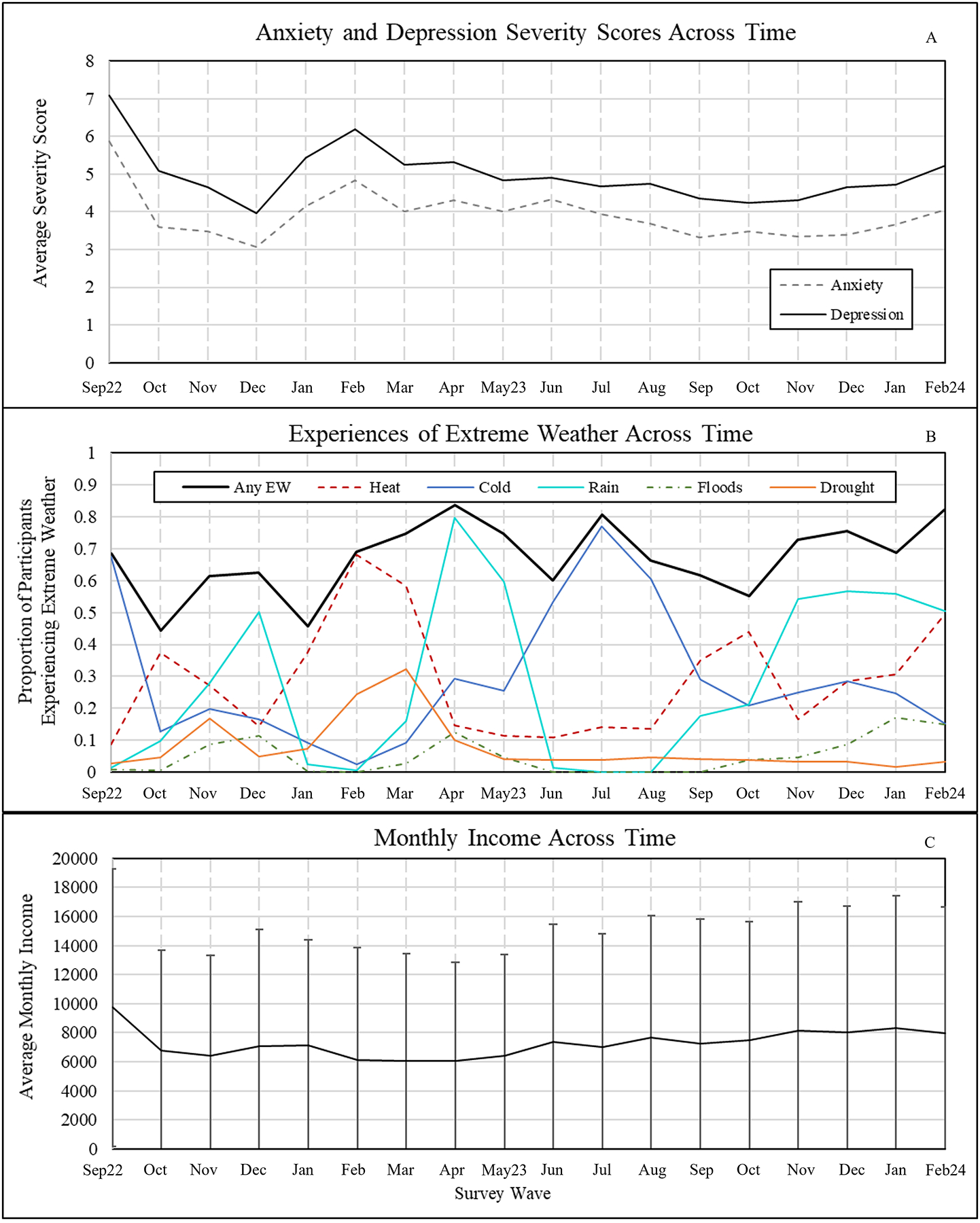
Anxiety, depression, experiences of extreme weather, and average monthly income across time.

Pooled average severity scores for anxiety and depression, proportion of the sample reporting experiences of each type of extreme weather, and average monthly income across time are illustrated in Fig 1.

Average anxiety and depression scores vary across time. Pooled estimates suggest they range between minimum = 3.1 (SD = 4.4) in wave three and maximum = 5.9 (SD = 5.1) at baseline for anxiety and minimum = 4.0 (SD = 5.0) in wave three and maximum = 7.1 (SD = 5.8) at baseline for depression. Average monthly incomes range from KES 6072 (SD = 7364) in wave six to KES 9731 (SD = 9556) at baseline. Actual values of reported monthly incomes range from KES 0 to KES 75,750. Experiences of extreme weather vary across time, with the most extreme heat events reported in wave five (February), the most extreme cold events in wave 10 (July), the most downpours in wave seven (April), the most flooding events in wave 16 (January), and the most drought events in wave six (March).

The intraclass correlation coefficients (ICCs) for anxiety and depression were 0.434 and 0.432, respectively, indicating that about 43% of the total variance was attributable to between-person differences. This suggests that some participants had relatively stable levels of anxiety and depression over time. The remaining ~57% of variance reflected within-person fluctuations, which suggests that some participants’ anxiety and depression scores are more reactive to changing conditions across time.

### Associations between experiences of extreme weather and anxiety and depression

Results from random slopes models examining associations between experiences of extreme weather and anxiety and depression are summarized in Tables 2 and 3, respectively. Experiencing any type of extreme weather was associated with a 0.56-point increase in symptoms of anxiety [95% CI (0.41, 0.71), p = .000] and a 0.44-point increase in symptoms of depression [95% CI (0.27, 0.61), p = .000]. Experiencing extreme heat was associated with a 0.24-point increase in symptoms of anxiety [95% CI (0.10, 0.38), p = .001] and a 0.36-point increase in symptoms of depression [95% CI (0.20, 0.53), p = .000]. Experiencing extreme cold was associated with a 0.50-point increase in symptoms of anxiety [95% CI (0.36, 0.64), p = .000] and a 0.53-point increase in symptoms of depression [95% CI (0.37, 0.69), p = .000]. Experiencing heavy rain was associated with a 0.19-point decrease in symptoms of anxiety [95% CI (−0.33, −0.05), p = .006] and a 0.24-point decrease in symptoms of depression [95% CI (−0. 39, −0.08), p = .003]. Experiencing flooding was not significantly associated with symptoms of anxiety or depression. Reports of flooding were infrequent. There were only four months during which more than 10% of the sample reported experiencing a flood. Finally, experiencing drought was associated with a 0.63-point increase in symptoms of anxiety [95% CI (0.38, 0.88), p = .000] and a 1.12-point increase in symptoms of depression [95% CI (0.84, 1.41), p = .000].

### Moderating effects of monthly income

The moderating effects of monthly income on experiences of extreme weather and anxiety and depression are summarized in Tables 4 and 5, respectively. Monthly income significantly moderated the relationship between anxiety and depression and experiencing any type of extreme weather, heat, and drought, but not for extreme cold, heavy rainfall, or flooding. For participants who experienced extreme heat, each increase of KES 1,000 was associated with a 0.03-point decrease in symptoms of anxiety [95% CI (−0.04, −0.01), p = .007] and a 0.02-point decrease in symptoms of depression [95% CI (−0.04, −0.003), p = .027]. For participants who experienced drought, each increase of KES 1,000 was associated with a 0.05-point decrease in symptoms of anxiety [95% CI (−0.09, −0.02), p = .006].

### Income thresholds to remove significant mental health effects of extreme weather

**Fig 2.**
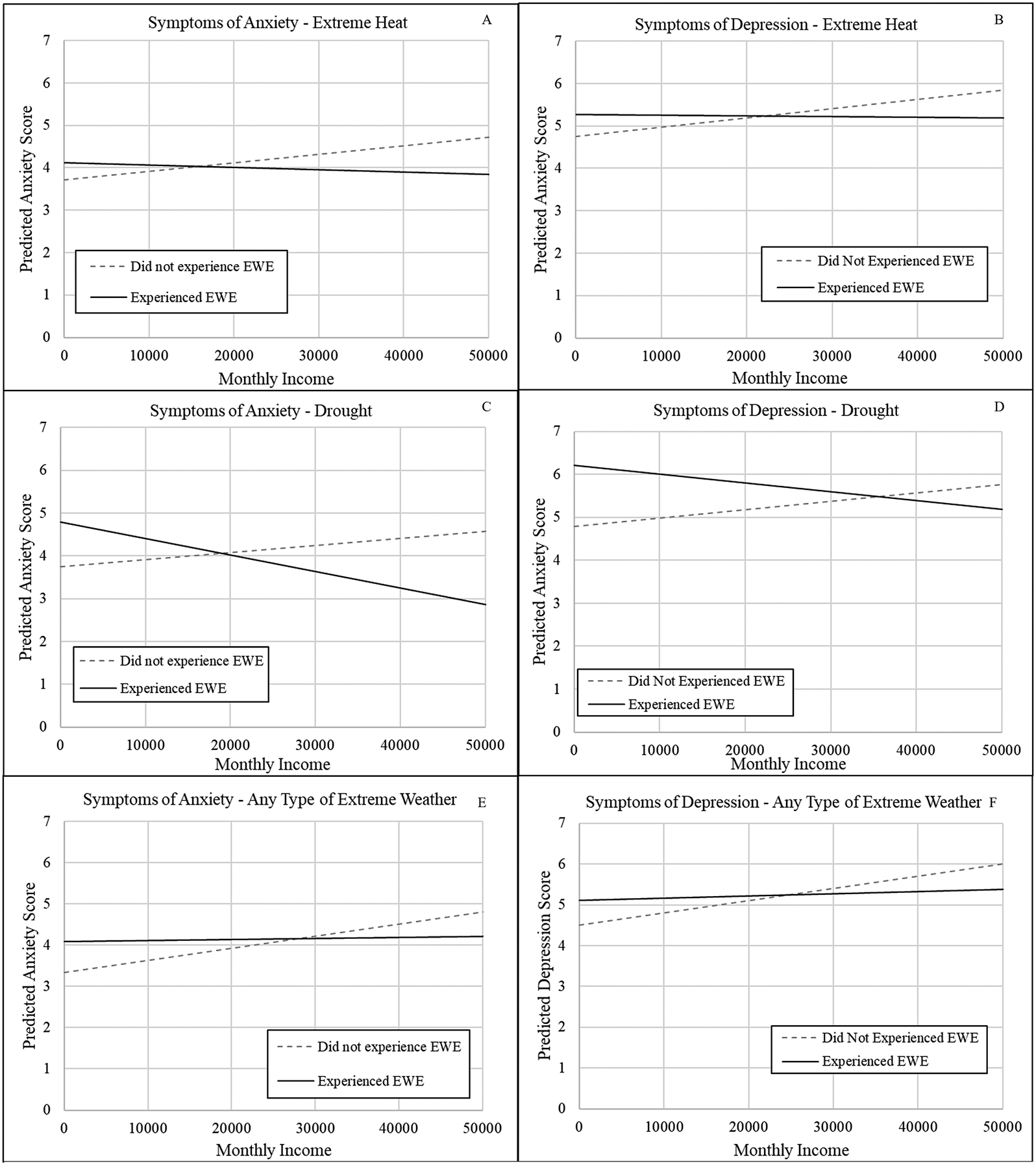
Predicted anxiety and depression scores across income levels for individuals with and without exposure to heat (A-B), drought (C-D), and any extreme weather (E-F).

Predicted values for models with significant income moderation are plotted in Fig 2. Findings show that for women who experienced extreme heat, average anxiety scores decreased from 4.1 to 3.8 as income increased, from KES 1,000 to KES 50,000. Similarly, depression scores decreased from 5.3 to 5.1 as income increased from KES 1,000 to KES 50,000. For participants who experienced drought, average anxiety scores decreased from 4.8 to 2.9 as income increased from KES 1,000 to KES 50,000.

Results from the marginal effects models are summarized in Table 6. These findings highlight thresholds related to the moderating effect of income on the relationships between extreme weather and symptoms of anxiety and depression. For example, findings suggest that for individuals that earned less than KES 11,000 (~US$87) per month, experiencing extreme heat was associated with a statistically detectable increase in anxiety symptoms; however, for those with incomes above this threshold, experiencing extreme heat was not associated with a statistically significant increase in anxiety symptoms. Similarly, for individuals earning less than KES 14,000 (~US$110) per month, experiencing extreme heat was associated with a statistically significant increase in depression symptoms, but above that income threshold, experiencing extreme heat did not have a statistically significant effect on depression symptoms.

For individuals earning less than KES 13,000 (~US$102), experiencing drought was associated with a statistically detectable increase in anxiety symptoms, but for individuals with monthly incomes above that level, experiencing drought was not associated with a statistically significant increase in anxiety symptoms.

## Discussion

Our findings indicate that experiences of extreme weather, including heat, cold, and drought, are significantly associated with increased symptoms of anxiety and depression. Heavy rain, in contrast, was associated with a decrease in these symptoms, while flooding showed no significant relationship. These findings partially align with past research linking extreme weather to poor mental health outcomes (2–5). However, our findings that heavy rainfall was associated with a decrease in mental health symptoms, and that flooding was not significantly associated, differed from earlier studies.

The lack of significance for flooding may be due to low exposure levels in our sample. Due to informality, structures are often built in riparian zones [91] within 30 meters of a rivers’ edge, where flood risk is greatest. In fact, because of this risk, residents in these zones pay lower rents. Only about 14% of the women in our study lived within 30 meters of a river at any point during the study, limiting our ability to detect flood-related effects. Future research should consider oversampling these high-risk areas.

The finding that heavy rainfall was associated with decreased symptoms of anxiety and depression could be attributed to numerous factors. The potential benefits of rain for women in informal settlements could be one such factor. Many rely on water for both unpaid domestic work and paid labor, including handwashing clothes, cleaning homes, or preparing food [6,13,14,40,60,61,92,93]. Over 86% of women in our study must purchase water, making free rainwater a valuable resource. Research suggests that, for many households in informal settlements, monthly expenditures exceed incomes [63], but harvesting rainwater may lessen this deficit and reduce the time women spend collecting water [56]. As one woman in this study stated, “*You can collect rain water and use it for washing as well as doing other cleaning with it. Then the one you buy, you use for cooking and drinking alone. Now that’s why you can spend less money when it’s raining.”*

This may be especially beneficial for women who farm, raise livestock, sell produce, or wash clothes.

Rains may also reduce financial stress, indirectly, by contributing to national food production—increasing food availability and lowering prices in informal settlements, where most residents must purchase food rather than produce it. On the other hand, parallel qualitative research suggests that heavy rains may cause flooding or property damage [56]. Additionally, rainwater quality is often poor, and leaks during heavy rains can affect physical health, sleep, and material possessions [56]. Despite this, women may value the financial benefits of rain over the physical costs. Further research is needed to weigh these tradeoffs.

We also found that monthly income moderated the effects of extreme weather exposure on mental health. Specifically, income buffered the effects of extreme heat, drought and overall exposure to extreme weather. During drought and heat, the cost of essential goods such as food and water often rise due to resource scarcity, disproportionately burdening residents of informal settlements [56]. Water rationing, for example, increases the time, effort, and cost required to access water. Women who cannot wait in longer queues or travel to alternative sources may be forced to buy water at prices 10–100 times higher than usual. Higher household income likely mitigates these burdens by enabling women to better adapt during climate extremes. Conversely, income was less influential during cold or rain events, perhaps, because these events coincide with seasons in Kenya when food and water are more readily available and, therefore, less costly.

Income thresholds based on marginal effects from the models offer important insights for policy and intervention. First, although the following income thresholds may not be generalized to other informal settlements, globally, the approach we used to identify them could be applied elsewhere to guide community-centered financial strategies that reduce climate-related health burdens in vulnerable settlements. For example, women with monthly incomes below KES 11,000 (~US$87) experienced statistically significant increases in anxiety when exposed to extreme heat; women with incomes below KES 13,000 (~US$102) experienced statistically significant increases in anxiety when exposed to drought. At higher incomes, these associations disappeared. This suggests income is particularly protective for women experiencing heat or drought with limited financial resources and less important for those in households with higher incomes. These findings suggest targeted financial interventions may protect women’s mental health during climate extremes.

Programs that raise households’ overall monthly incomes, especially for those earning below KES 19,000 (~US$150), could reduce climate-related mental health burdens. For example, government initiatives like Kazi Mtaani/Climate WorX or the National Youth Service’s Slum Improvement Initiative in Kenya [74], have provided vulnerable populations, such as youth and women, with paid opportunities to improve local infrastructure and waste management and address environmental challenges in informal settlements [74]. While criticized for low pay and limited access, expanding and sustaining such programs could address financial vulnerability while simultaneously improving ecological resilience.

Finance-based forecasting and household-level climate resilience grants offer other promising avenues. By issuing cash transfers in anticipation of forecasted weather extremes, these programs help vulnerable households adapt proactively. However, thresholds for issuing payments must be focused on and tailored to climate-vulnerable communities. Due to their ecological exposure and social marginalization, informal settlement residents often experience adverse effects at lower thresholds than the general population [78]. Thus, centering the experiences of these communities and co-developing context-specific triggers for cash payments is crucial for program success. Alternatively, or in tandem with cash transfers, larger, one-time climate resilience grants for households in climate-vulnerable communities could help buffer the mental health impacts of climate change. Many residents, especially women, lack finances to move to less vulnerable homes, e.g., away from rivers, or invest in in-situ climate resilience strategies such as home improvements, water and sanitation investments, food storage, urban agriculture, solar energy, etc.

Thus, women’s empowerment should also be central to strategies that address the mental health impacts of climate-related shocks. Empowering women through inclusive decision-making and governance can enhance both equity and effectiveness in climate adaptation [94,95]. Women-led initiatives also foster economic stability and community cohesion [75]. Their local knowledge and roles within households, communities, and resource management make them critical to addressing climate risks [19,75,94,96].

Informal settlements often receive fewer public resources and limited institutional support, leaving residents, particularly women, to rely on alternative forms of social and financial organization to manage adversity and build climate resilience [10,60,75,95,97,98]. Women’s self-help groups (SHGs) and table banking initiatives, for example, have evolved as trusted mechanisms for savings, mutual support, and collective action in informal settlements, and can serve as vital platforms for enhancing climate resilience [99,100]. Support for these groups can be expanded to establish savings and contingency funds that help women buffer against income losses and livelihood shocks during EWEs. Federations, or collectives of these savings groups, can also push for larger-scale action like tenure security and settlement upgrading, which build climate resilience in informal settlements [10,97]. Strengthening such collective financial mechanisms could help reduce stress, anxiety, and feelings of helplessness during periods of crisis by improving women’s sense of control and security over household resources. Government bodies such as the National Government Affirmative Action Fund (NGAAF) in Kenya can further strengthen and expand empowerment programs that target women in vulnerable communities, ensuring that climate adaptation and mental health interventions are inclusive, gender-responsive, and sustainable.

Although this paper presents important findings, several limitations should be acknowledged. First, we relied on self-reported experiences of extreme weather rather than meteorological data. Although this was an active choice we made because some research suggests it better captures subjective experience, it can be vulnerable to recall bias, especially for gradual events like heat or cold [78]. Future research could work with communities to iteratively refine community-specific definitions of “extreme” using both self-report and meteorological data. Second, we gathered data from one woman per household, and 7% were unable to report household finances. Collecting data from multiple members of the household may improve future estimates. Third, reported monthly incomes in this sample were low, with a maximum of KES 75,750 and few data points above KES 50,000. While this is likely an accurate reflection of women’s limited access to financial resources in informal settlements, this limited variability made it hard to extrapolate potential buffering effects at higher incomes. Fourth, there is a complex nexus of social and environmental factors that influence mental health in highly dynamic urban informal settings [46]. Although we controlled for IPV and food insecurity in our mixed-effects models—two major correlates of mental distress [24,86]—other contextual factors that were not measured in this study, such a food prices, could act as confounders or mediators. Continued work is needed to isolate causal pathways and identify modifiable targets for intervention. Finally, climate exposure can vary between informal settlements. While the process we used to determine critical income thresholds to help protect residents, especially women, from poor mental health could be applied in other informal settlements, globally, the specific thresholds we present may not be generalizable.

## Conclusion

Overall, this study provides important longitudinal evidence of the relationship between experiences of extreme weather and women’s symptoms of anxiety and depression in Nairobi’s informal settlements, revealing that the mental health impacts of weather extremes vary by type. While heat, cold, and drought are associated with increased symptoms of anxiety and depression, heavy rain appears to be associated with a reduction in symptoms—perhaps due to its financial and time-saving benefits for women reliant on water for domestic and income-generating activities. Findings also suggest that monthly income is an important moderating factor for the mental health impacts of heat and drought, with lower-income women experiencing the greatest burden. This is a critical climate justice issue given that the residents who have the fewest financial resources to adapt to extreme weather are the ones whose mental health is most affected. Targeted, income-based interventions—such as sustainable employment programs, finance-based forecasting, and climate resilience grants—could help mitigate the negative effects of climate extremes in informal settlements and, in doing so, start to address a critical equity issue. Furthermore, while the specific income thresholds found in this study may not be generalizable to other informal settlements, the process for determining key thresholds could be applied in other settings—helping to establish community-centered parameters for finance-based solutions to reduce the health burden of climate change in especially vulnerable communities. We emphasize that interventions should be co-produced with residents of climate-vulnerable communities to ensure they reflect local needs and experiences rather than reinforcing the inequities common in top-down approaches. Future research should focus on high-risk subgroups, explore additional health outcomes beyond mental health, empower women, and engage communities in co-developing context-sensitive adaptation strategies.

## Supplementary Material

Supplemental Materials

S1 Checklist. Inclusivity in global health research.

(PDF)

## Figures and Tables

**Table 1.** Descriptive statistics for time-invariant variables (n_baseline_ = 800).

Covariate	Median(IQR) or Pooled Proportion
Age	34 (27–42)
Education	
Less than primary	25.0
Completed primary	47.0
Completed secondary	28.0
Married	55.0
Settlement	
Kibera	50.0
Mathare	50.0
Survey Wave	
0 (baseline)	100.0
1	97.9
2	96.6
3	95.5
4	96.0
5	95.1
6	94.4
7	88.4
8	92.3
9	92.3
10	91.6
11	93.1
12	90.5
13	92.3
14	87.8
15	89.8
16	89.5
17	88.3

**Table 2.** Associations between experiencing extreme weather and symptoms of anxiety.

	Any	Heat	Cold	Rain	Flooding	Drought
Experienced EWE (ref: did not experience)	0.56*** [0.41–0.71]	0.24** [0.098–0.381]	0.5*** [0.359–0.64]	−0.19** [−0.329–0.054]	0.21 [−0.067–0.492]	0.63*** [0.379–0.881]
Monthly income (in 1,000s)	0.014** [0.004–0.024]	0.014** [0.004–0.024]	0.012* [0.002–0.022]	0.013** [0.003–0.023]	0.014** [0.004–0.023]	0.014** [0.004–0.024]
Education (ref: less than primary)						
Completed primary	−0.24 [−0.732–0.246]	−0.24 [−0.739–0.25]	−0.24 [−0.735–0.249]	−0.25 [−0.749–0.249]	−0.25 [−0.745–0.25]	−0.24 [−0.734–0.256]
Completed secondary	−0.97** [−1.527–0.405]	−0.99** [−1.557–0.423]	−0.98** [−1.546–0.417]	−1.00** [−1.573–0.43]	−0.99** [−1.565–0.424]	−0.98** [−1.552–0.416]
Partner works	−0.92*** [−1.127–0.706]	−0.92*** [−1.126–0.704]	−0.93*** [−1.138–0.717]	−0.91*** [−1.126–0.704]	−0.92*** [−1.13–0.708]	−0.91*** [−1.118–0.697]
Participant works	−0.44*** [−0.643–0.231]	−0.42*** [−0.629–0.216]	−0.42*** [−0.626–0.214]	−0.41*** [−0.618–0.206]	−0.42*** [−0.628–0.215]	−0.42*** [−0.629–0.217]
Married	−0.01 [−0.313–0.284]	−0.02 [−0.316–0.284]	−0.003 [−0.303–0.297]	−0.02 [−0.318–0.284]	−0.01 [−0.316–0.286]	−0.02 [−0.32–0.281]
Lives in Mathare (ref: Lives in Kibera)	0.9*** [0.527–1.279]	0.91*** [0.535–1.294]	0.94*** [0.557–1.313]	0.92*** [0.535–1.3]	0.92*** [0.534–1.297]	0.86*** [0.484–1.246]
Age	0.03** [0.011–0.044]	0.03** [0.01–0.043]	0.03** [0.011–0.044]	0.03** [0.009–0.043]	0.03** [0.009–0.043]	0.03** [0.009–0.043]
Any IPV	1.08*** [0.871–1.29]	1.1*** [0.893–1.313]	1.09*** [0.877–1.296]	1.11*** [0.896–1.316]	1.11*** [0.898–1.317]	1.09*** [0.881–1.3]
Food insecurity	0.14*** [0.132–0.156]	0.15*** [0.134–0.158]	0.14*** [0.131–0.155]	0.15*** [0.134–0.157]	0.15*** [0.134–0.158]	0.15*** [0.134–0.158]
Survey timepoint	−0.04*** [−0.06–0.025]	−0.04*** [−0.056–0.021]	−0.04*** [−0.057–0.022]	−0.03*** [−0.052–0.017]	−0.04*** [−0.056–0.021]	−0.03*** [−0.052–0.017]
Random slope SD (individual differences in change)	0.19 [0.177–0.208]	0.19 [0.179–0.21]	0.19 [0.18–0.211]	0.19 [0.18–0.21]	0.19 [0.179–0.209]	0.19 [0.179–0.21]
Random intercept SD (between-person variance)	2.48 [2.324–2.644]	2.51 [2.356–2.679]	2.5 [2.341–2.663]	2.54 [2.382–2.705]	2.53 [2.375–2.698]	2.52 [2.36–2.682]
Within-person (residual) SD	3.29 [3.246–3.331]	3.29 [3.248–3.333]	3.29 [3.243–3.328]	3.29 [3.246–3.331]	3.29 [3.248–3.333]	3.29 [3.245–3.33]
ICC	0.434					

Notes: Results reflect six separate mixed-effects models, each regressing anxiety on a different extreme weather exposure. Coefficients represent adjusted beta estimates with 95% confidence intervals in brackets.

Statistical significance is denoted as *** p < .001, ** p < .01, * p < .05, † p < .10. Reference category for categorical variables is indicated in parentheses.

**Table 3.** Associations between experiencing extreme weather and symptoms of depression.

Depression	Any	Heat	Cold	Rain	Flooding	Drought
Experienced EWE (ref: did not experience)	0.44*** [0.265–0.61]	0.36*** [0.202–0.527]	0.53*** [0.365–0.686]	−0.24** [−0.394–0.079]	0.3† [−0.018–0.623]	1.12*** [0.835–1.41]
Monthly income (in 1,000s)	0.016** [0.005–0.027]	0.016** [0.005–0.028]	0.014* [0.003–0.025]	0.015** [0.004–0.026]	0.016** [0.004–0.027]	0.017** [0.006–0.028]
Education (ref: less than primary)						
Completed primary	−0.13 [−0.707–0.441]	−0.13 [−0.706–0.443]	−0.13 [−0.705–0.443]	−0.14 [−0.717–0.44]	−0.14 [−0.714–0.442]	−0.12 [−0.697–0.456]
Completed secondary	−1.03** [−1.689–0.367]	−1.04** [−1.703–0.379]	−1.04** [−1.697–0.375]	−1.06** [−1.723–0.391]	−1.05** [−1.714–0.383]	−1.03** [−1.693–0.366]
Partner works	[−1.339–0.851]	−1.09*** [−1.338–0.851]	−1.11*** [−1.351–0.863]	−1.09*** [−1.338–0.851]	−1.1*** [−1.342–0.854]	−1.08*** [−1.324–0.838]
Participant works	−0.62*** [−0.861–0.385]	−0.61*** [−0.853–0.377]	−0.61*** [−0.848–0.373]	−0.6*** [−0.839–0.363]	−0.61*** [−0.851–0.375]	−0.62*** [−0.855–0.379]
Married	0.25 [−0.095–0.596]	0.25 [−0.093–0.599]	0.26 [−0.081–0.61]	0.25 [−0.095–0.598]	0.25 [−0.094–0.599]	0.25 [−0.1–0.592]
Lives in Mathare (ref: Lives in Kibera)	1.27*** [0.833–1.709]	1.27*** [0.834–1.712]	1.3*** [0.865–1.741]	1.28*** [0.838–1.721]	1.28*** [0.838–1.72]	1.18*** [0.74–1.621]
Age	0.05*** [0.033–0.072]	0.05*** [0.032–0.071]	0.05*** [0.033–0.072]	0.05*** [0.032–0.071]	0.05*** [0.032–0.071]	0.05*** [0.031–0.07]
Any IPV	1.06*** [0.816–1.299]	1.07*** [0.832–1.314]	1.06*** [0.816–1.298]	1.08*** [0.837–1.32]	1.08*** [0.839–1.321]	1.05*** [0.808–1.29]
Food insecurity	0.16*** [0.146–0.173]	0.16*** [0.148–0.175]	0.16*** [0.144–0.172]	0.16*** [0.147–0.174]	0.16*** [0.148–0.175]	0.16*** [0.147–0.175]
Survey timepoint	−0.05*** [−0.074–0.032]	−0.05*** [−0.071–0.029]	−0.05*** [−0.072–0.029]	−0.04*** [−0.066–0.024]	−0.05*** [−0.071–0.029]	−0.04*** [−0.064–0.022]
Random slope SD (individual differences in change)	0.24 [0.219–0.255]	0.24 [0.218–0.255]	0.24 [0.221–0.257]	0.24 [0.219–0.256]	0.24 [0.219–0.256]	0.24 [0.218–0.255]
Random intercept SD (between-person variance)	2.9 [2.719–3.094]	2.91 [2.724–3.099]	2.9 [2.717–3.091]	2.93 [2.749–3.125]	2.93 [2.744–3.12]	2.92 [2.735–3.11]
Within-person (residual) SD	3.76 [3.715–3.813]	3.76 [3.716–3.814]	3.76 [3.712–3.81]	3.76 [3.715–3.813]	3.77 [3.716–3.814]	3.76 [3.71–3.807]
ICC	0.432					

Notes: Results reflect six separate mixed-effects models, each regressing depression on a different extreme weather exposure. Coefficients represent adjusted beta estimates with 95% confidence intervals in brackets.

Statistical significance is denoted as *** p < .001, ** p < .01, * p < .05, † p < .10. Reference category for categorical variables is indicated in parentheses.

**Table 4.** Moderation effects of monthly income on experiencing extreme weather and symptoms of anxiety.

Anxiety	Any	Heat	Cold	Rain	Flooding	Drought
Experienced EWE (ref: did not experience)	0.74*** [0.557–0.931]	0.42*** [0.225–0.606]	0.56*** [0.372–0.754]	−0.22* [−0.409–0.038]	0.14 [−0.271–0.545]	1.01*** [0.639–1.375]
Monthly income (in 1,000s)	0.029*** [0.016–0.043]	0.02*** [0.009–0.031]	0.015** [0.004–0.026]	0.012* [0.002–0.023]	0.013** [0.003–0.023]	0.017** [0.007–0.027]
Experienced EWE*Montly Income (in 1,000s)	−0.027** [−0.043–0.011]	−0.026** [−0.044–0.007]	−0.009 [−0.026–0.009]	0.005 [−0.014–0.023]	0.01 [−0.028–0.047]	−0.052** [−0.089–0.015]
Education (ref: less than primary)						
Completed primary	−0.24 [−0.729–0.25]	−0.24 [−0.739–0.249]	−0.24 [−0.735–0.25]	−0.25 [−0.748–0.249]	−0.25 [−0.745–0.25]	−0.23 [−0.726–0.264]
Completed secondary	−0.97** [−1.528–0.405]	−0.99** [−1.559–0.426]	−0.98** [−1.546–0.417]	−1.00** [−1.572–0.428]	−0.99** [−1.564–0.423]	−0.98** [−1.549–0.413]
Partner works	−0.91*** [−1.121–0.701]	−0.92*** [−1.128–0.706]	−0.93*** [−1.137–0.716]	−0.92*** [−1.126–0.704]	−0.92*** [−1.13–0.708]	−0.91*** [−1.118–0.697]
Participant works	−0.44*** [−0.643–0.231]	−0.42*** [−0.623–0.21]	−0.42*** [−0.628–0.216]	−0.41*** [−0.617–0.204]	−0.42*** [−0.627–0.214]	−0.42*** [−0.625–0.213]
Married	−0.02 [−0.315–0.283]	−0.01 [−0.311–0.289]	−0.004 [−0.304–0.296]	−0.02 [−0.317–0.285]	−0.01 [−0.315–0.286]	−0.02 [−0.321–0.28]
Lives in Mathare (ref: Lives in Kibera)	0.9*** [0.528–1.281]	0.91*** [0.529–1.288]	0.94*** [0.557–1.313]	0.92*** [0.535–1.3]	0.92*** [0.535–1.299]	0.86*** [0.477–1.239]
Age	0.03** [0.011–0.044]	0.03** [0.01–0.043]	0.03** [0.011–0.044]	0.03** [0.009–0.043]	0.03** [0.009–0.043]	0.03** [0.009–0.043]
Any IPV	1.08*** [0.874–1.293]	1.11*** [0.898–1.317]	1.09*** [0.877–1.296]	1.11*** [0.897–1.316]	1.11*** [0.897–1.317]	1.09*** [0.883–1.302]
Food insecurity	0.14*** [0.132–0.156]	0.15*** [0.134–0.158]	0.14*** [0.131–0.155]	0.15*** [0.134–0.157]	0.15*** [0.134–0.158]	0.15*** [0.134–0.158]
Survey timepoint	−0.04*** [−0.061–0.025]	−0.04*** [−0.056–0.02]	−0.04*** [−0.057–0.022]	−0.03*** [−0.053–0.017]	−0.04*** [−0.057–0.021]	−0.04*** [−0.053–0.017]
Random slope SD (individual differences in change)	0.19 [0.177–0.208]	0.19 [0.178–0.209]	0.19 [0.18–0.211]	0.19 [0.179–0.21]	0.19 [0.179–0.209]	0.19 [0.179–0.21]
Random intercept SD (between-person variance)	2.48 [2.327–2.647]	2.51 [2.353–2.675]	2.5 [2.342–2.663]	2.54 [2.382–2.705]	2.53 [2.376–2.699]	2.52 [2.361–2.683]
Within-person (residual) SD	3.29 [3.244–3.329]	3.29 [3.247–3.332]	3.28 [3.243–3.328]	3.29 [3.246–3.331]	3.29 [3.248–3.333]	3.29 [3.244–3.329]

Notes: Results reflect six separate mixed-effects models, each regressing anxiety on a different extreme weather exposure. Models include an interaction term with income to test moderation. Coefficients represent adjusted beta estimates with 95% confidence intervals in brackets.

Statistical significance is denoted as *** p < .001, ** p < .01, * p < .05, † p < .10. Reference category for categorical variables is indicated in parentheses.

**Table 5.** Moderation effects of monthly income on experiencing extreme weather and symptoms of depression.

Depression	Any	Heat	Cold	Rain	Flooding	Drought
Experienced EWE (ref: did not experience)	0.61*** [0.393–0.821]	0.53*** [0.31–0.742]	0.63*** [0.411–0.851]	−0.23* [−0.44–0.014]	0.49* [0.021–0.957]	1.41*** [1.00–1.826]
Monthly income (in 1,000s)	0.03*** [0.015–0.045]	0.022*** [0.01–0.034]	0.018** [0.005–0.031]	0.016* [0.004–0.027]	0.017** [0.005–0.028]	0.019** [0.008–0.031]
Experienced EWE*Monthly Income (in 1,000s)	−0.025* [−0.043–0.006]	−0.024* [−0.044–0.003]	−0.014 [−0.035–0.006]	−0.001 [−0.023–0.020]	−0.024 [−0.067–0.020]	−0.04† [−0.081–0.001]
Education (ref: less than primary)						
Completed primary	−0.13 [−0.704–0.444]	−0.13 [−0.706–0.443]	−0.13 [−0.705–0.443]	−0.14 [−0.717–0.44]	−0.14 [−0.714–0.442]	−0.11 [−0.69–0.462]
Completed secondary	−1.03** [−1.69–0.367]	−1.04** [−1.706–0.382]	−1.04** [−1.697–0.375]	−1.06** [−1.724–0.391]	−1.05** [−1.717–0.387]	−1.03** [−1.691–0.364]
Partner works	−1.09*** [−1.335–0.847]	[−1.34–0.853]	−1.11*** [−1.349–0.861]	−1.09*** [−1.338–0.851]	[−1.342–0.854]	−1.08*** [−1.324–0.837]
Participant works	−0.62*** [−0.862–0.385]	−0.61*** [−0.847–0.371]	−0.61*** [−0.851–0.375]	−0.6*** [−0.839–0.363]	−0.62*** [−0.853–0.377]	−0.61*** [−0.852–0.376]
Married	0.25 [−0.095–0.596]	0.26 [−0.089–0.602]	0.26 [−0.082–0.608]	0.25 [−0.095–0.598]	0.25 [−0.095–0.598]	0.25 [−0.1–0.591]
Lives in Mathare (ref: Lives in Kibera)	1.27*** [0.833–1.709]	1.27*** [0.829–1.706]	1.3*** [0.864–1.74]	1.28*** [0.838–1.721]	1.28*** [0.835–1.717]	1.18*** [0.735–1.616]
Age	0.05*** [0.033–0.071]	0.05*** [0.032–0.071]	0.05*** [0.033–0.072]	0.05*** [0.032–0.071]	0.05*** [0.032–0.071]	0.05*** [0.031–0.07]
Any IPV	1.06*** [0.819–1.301]	1.08*** [0.836–1.318]	1.06*** [0.817–1.299]	1.08*** [0.837–1.32]	1.08*** [0.839–1.322]	1.05*** [0.81–1.292]
Food insecurity	0.16*** [0.146–0.174]	0.16*** [0.148–0.175]	0.16*** [0.144–0.172]	0.16*** [0.147–0.174]	0.16*** [0.148–0.176]	0.16*** [0.147–0.174]
Survey timepoint	−0.05*** [−0.074–0.032]	−0.05*** [−0.07–0.029]	−0.05*** [−0.072–0.03]	−0.04*** [−0.066–0.024]	−0.05*** [−0.071–0.029]	−0.04*** [−0.064–0.023]
Random slope SD (individual differences in change)	0.24 [0.218–0.255]	0.24 [0.218–0.254]	0.24 [0.22–0.257]	0.24 [0.219–0.256]	0.24 [0.219–0.256]	0.24 [0.218–0.255]
Random intercept SD (between-person variance)	2.90 [2.721–3.096]	2.90 [2.722–3.097]	2.90 [2.718–3.093]	2.93 [2.749–3.125]	2.92 [2.743–3.118]	2.92 [2.735–3.11]
Within-person (residual) SD	3.76 [3.715–3.812]	3.76 [3.716–3.814]	3.76 [3.712–3.809]	3.76 [3.715–3.813]	3.76 [3.716–3.814]	3.76 [3.709–3.807]

Notes: Results reflect six separate mixed-effects models, each regressing depression on a different extreme weather exposure. Models include an interaction term with income to test moderation. Coefficients represent adjusted beta estimates with 95% confidence intervals in brackets.

Statistical significance is denoted as *** p < .001, ** p < .01, * p < .05, † p < .10. Reference category for categorical variables is indicated in parentheses.

**Table 6.** Income thresholds affecting relationship between experiencing extreme weather and symptoms of anxiety and depression.

Type of Extreme	Anxiety		Depression	
Weather	Thresholds	Effect and Significance	Thresholds	Effect and Significance
Heat	Below 11,000	Statistically detectable increase in anxiety symptoms associated with experiencing extreme weather	Below 14,000	Statistically detectable increase in depressive symptoms associated with experiencing extreme weather
	Above 11,000	No statistically detectable change in anxiety symptoms associated with experiencing extreme weather	Above 14,000	No statistically detectable change in depressive symptoms associated with experiencing extreme weather
Drought	Below 13,000	Statistically detectable increase in anxiety symptoms associated with experiencing extreme weather	Below 21,000	Statistically detectable increase in depressive symptoms associated with experiencing extreme weather
	Above 13,000	No statistically detectable change in anxiety symptoms associated with experiencing extreme weather	Above 21,000	No statistically detectable change in depressive symptoms associated with experiencing extreme weather
Any	Below 19,000	Statistically detectable increase in anxiety symptoms associated with experiencing extreme weather	Below 16,000	Statistically detectable increase in depressive symptoms associated with experiencing extreme weather
	Above 19,000	No statistically detectable change in anxiety symptoms associated with experiencing extreme weather	Above 16,000	No statistically detectable change in depressive symptoms associated with experiencing extreme weather

## Data Availability

All deidentified data, code, and documentation relevant to this paper are available in the Zenodo repository at DOI: [https://doi.org/10.5281/zenodo.17822614]. No additional restrictions apply.
